# AN EXAMINATION OF STAFF SELF-EFFICACY MEASUREMENT IN THE PALLIATIVE CARE EDUCATION IN ASSISTED LIVING INTERVENTION

**DOI:** 10.1093/geroni/igae098.0928

**Published:** 2024-12-31

**Authors:** Debra Dobbs, Jessica Yauk, Hongdao Meng, William Haley, Lindsay Peterson, Harleah Buck

**Affiliations:** University of South Florida, Tampa, Florida, United States; University of South Florida, Tampa, Florida, United States; University of South Florida, Tampa, Florida, United States; University of South Florida, Tampa, Florida, United States; University of South Florida, Tampa, Florida, United States; University of Iowa, Iowa city, Iowa, United States

## Abstract

Advance care planning (ACP) is a necessary first step to meeting care preferences at the end of life and an important aspect of palliative care provision. ACP in assisted living (AL) is lacking for older adults living with dementia. A palliative care educational intervention found that only 12% of residents in AL living with dementia at baseline had documentation of (ACP) in their charts. Increased staff self-efficacy to have ACP discussions could potentially improve outcomes in ACP documentation. The current study is an NIH Stage 1b intervention using a sample of 23 nurses and administrators in 10 ALs to test if staff self-efficacy mediates the effects of increased documentation of ACP discussions. We estimated mediation models to examine whether staff self-efficacy (using a two-item scaled measure) mediates the relationship between the Palliative Care Education in Assisted Living intervention and ACP discussion rate. The PCEAL intervention had a positive impact on both staff self-efficacy and ACP discussion rate but the mediation results were not significant. In addition, an analysis of treatment fidelity ACP discussion checklists data showed the scores overall were lower than average (3.5 out of 7). We will discuss the differential scores between the self-reported increases from baseline to one-month (post-intervention) and lower than average observed treatment fidelity checklist scores of ACP Discussions by treatment staff and ways to improve the measurement of staff self-efficacy in future intervention research with staff who provide dementia care in assisted living settings.

